# Emerging roles of ATG7 in human health and disease

**DOI:** 10.15252/emmm.202114824

**Published:** 2021-11-02

**Authors:** Jack J Collier, Fumi Suomi, Monika Oláhová, Thomas G McWilliams, Robert W Taylor

**Affiliations:** ^1^ Wellcome Centre for Mitochondrial Research, Translational and Clinical Research Institute Newcastle University Newcastle upon Tyne UK; ^2^ Translational Stem Cell Biology & Metabolism Program, Research Programs Unit University of Helsinki Helsinki Finland; ^3^ Department of Anatomy Faculty of Medicine University of Helsinki Helsinki Finland; ^4^ NHS Highly Specialised Service for Rare Mitochondrial Disorders of Adults and Children Newcastle University Newcastle upon Tyne UK; ^5^ Present address: Department of Neurology and Neurosurgery Montreal Neurological Institute McGill University Montreal QC Canada

**Keywords:** ATG7, autophagy, disease, neurodegeneration, therapeutics, Autophagy & Cell Death

## Abstract

The cardinal stages of macroautophagy are driven by core autophagy‐related (ATG) proteins, whose ablation largely abolishes intracellular turnover. Disrupting ATG genes is paradigmatic of studying autophagy deficiency, yet emerging data suggest that ATG proteins have extensive biological importance beyond autophagic elimination. An important example is ATG7, an essential autophagy effector enzyme that in concert with other ATG proteins, also regulates immunity, cell death and protein secretion, and independently regulates the cell cycle and apoptosis. Recently, a direct association between ATG7 dysfunction and disease was established in patients with biallelic *ATG7* variants and childhood‐onset neuropathology. Moreover, a prodigious body of evidence supports a role for ATG7 in protecting against complex disease states in model organisms, although how dysfunctional ATG7 contributes to manifestation of these diseases, including cancer, neurodegeneration and infection, in humans remains unclear. Here, we systematically review the biological functions of ATG7, discussing the impact of its impairment on signalling pathways and human pathology. Future studies illuminating the molecular relationship between ATG7 dysfunction and disease will expedite therapies for disorders involving ATG7 deficiency and/or impaired autophagy.

## Graphical abstract

In this review, R. Taylor, J. Collier & colleagues discuss the biological functions of the autophagy protein ATG7, and the impact of its impairment on signalling pathways and human pathology

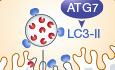

**Glossary**
Autophagic flux: The amount of autophagic degradation or activity that occurs over a specific time, typically referring to non‐selective (bulk) degradation or “macroautophagy”. Flux is typically measured by treating cells or tissues with various compounds that inhibit or activate autophagy. Autophagic turnover occurs at steady state within mammals.
Autophagosome: A transient double‐membrane‐bound organelle that sequesters cytoplasmic cargo and fuses with the endolysosomal system whose hydrolytic enzymes degrade its constituents.
Autophagy conjugation system: The group of proteins (including ATG3, ATG4, ATG5, ATG7, ATG10 and ATG12) that drive the lipidation of ATG8 homologues.
Autophagy: A homeostatic and developmental process that drives the delivery of damaged or unwanted cytoplasmic material to the endolysosomal system for degradation.
Biallelic: When both copies of an individual gene are affected by DNA variants.
Complex disorder: A disorder that cannot be explained by variants affecting a single gene. These are thought to be caused by the interactions between variants on a number of genes and environment.
Conditional knockout: When a gene and its protein are selectively eliminated or depleted from a specific tissue.
LC3‐associated phagocytosis: A process whereby extracellular material (e.g. a pathogen) is engulfed into an LC3‐positive single‐membrane structure that is delivered to the endolysosomal system for degradation.
LIR motif: The LC3‐interacting region (LIR) motif [(W/F/Y)XX(L/I/V)] is an amino acid sequence within proteins that enable them to interact directly with ATG8 homologues.
Recessive: Heritable characteristics that have an effect when a variant that controls the characteristic is present on both copies of a single gene. The same variant could be present on both alleles, or each allele could harbour a different variant that together has an effect.
Selective autophagy: Refers to a growing number of pathways that target specific cargo (e.g. mitochondria) for autophagic elimination. These pathways rely on specific adaptors that interact with both the cargo and autophagosome‐bound ATG8 homologues.

## Introduction

The degradation of encapsulated cytoplasmic material via the endolysosomal system provides a first‐principle definition of autophagy. Numerous specialised autophagy pathways have been discovered and categorised, including macroautophagy, which can be selective or non‐selective, and other variants such as chaperone‐mediated autophagy (CMA) and microautophagy. CMA recognises protein substrates with KFERQ motifs and translocates these to lysosomes (Kaushik & Cuervo, 2018). Microautophagy involves the direct engulfment and destruction of cytoplasmic substrates by lysosomal membrane invagination (Schuck, 2020). Macroautophagy (hereafter, “autophagy”) remains the most widely studied pathway. During autophagy, a transient double‐membrane‐bound autophagosome engulfs cytoplasmic constituents, eventually fusing with acidic endolysosomal compartments where hydrolysis degrades cargo (Yorimitsu & Klionsky, 2005).

**Figure 1:**
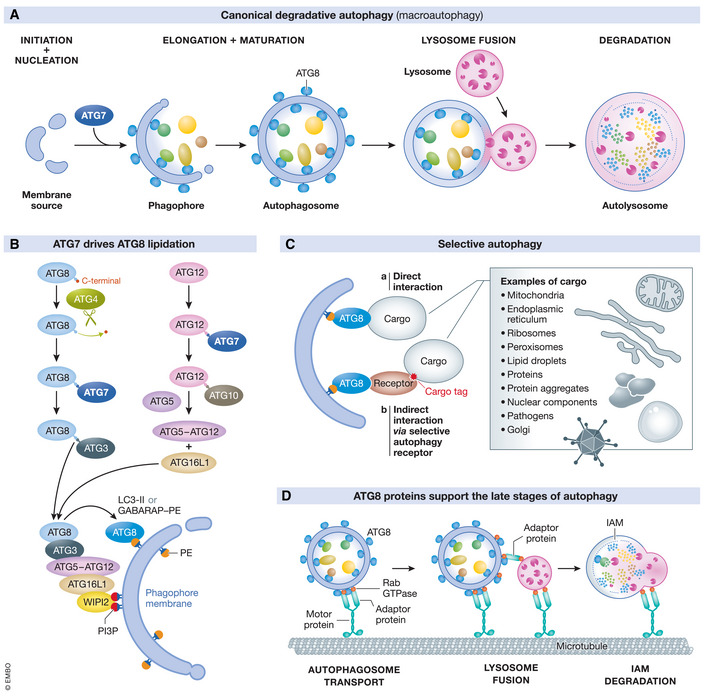
ATG7 drives the fundamental stages of degradative autophagy (A) An overview of classical degradative autophagy showing the early, middle and late stages of the process. (B) Phagophore expansion is stimulated by ATG7, which facilitates ATG8 lipidation through its E1‐like enzymatic activity. (C) ATG8 lipidation is also critical for selective autophagy and (D) contributes to the late stages of autophagy.

The fundamental morphological and molecular signatures of autophagy have remained largely unchanged over the past 10 years (Levine & Kroemer, 2008, 2019). Autophagy functions constitutively under basal conditions (Mizushima *et al*, 2004), but can be induced further by a number of stimuli, including starvation, hypoxia and DNA damage (Kroemer *et al*, 2010). This triggers the *de novo* nucleation of a phagophore, a double‐membrane cup‐shaped structure that matures via the incorporation of supplementary lipids (Nakamura & Yoshimori, 2017). A variety of cytoplasmic cargoes, including organelles, microbes and cytotoxic protein aggregates can be sequestered within this transient structure (Johansen & Lamark, 2020). Autophagy can be a non‐selective or selective process (Mizushima & Komatsu, 2011). “Bulk” autophagy involves the non‐selective sequestration of cytoplasmic material, ensuring the degradation of long‐lived proteins and replenishment of essential building blocks. During selective autophagy, specific cellular components are decorated with specialised signals that recruit the autophagic machinery to the target entity for elimination. Selectivity is conferred upon the pathway by LC3‐interacting region (LIR) motifs [(W/F/Y)XX(L/I/V)]) that are present within cargo or specialised adaptor proteins, enabling them to interact with ATG8 proteins that are embedded within the inner and outer membrane of the phagophore (Martens & Fracchiolla, 2020). Following cargo sequestration, the leading edges of the double‐membrane structure fuse to generate an autophagosome which merges with acidic compartments of the endolysosomal system. After the inner autophagosomal membrane is degraded, the resident lysosomal acid hydrolases degrade the autophagic cargo which is then recycled (Koyama‐Honda *et al*, 2017) (Fig 1A).

In the 1990s, pioneering studies using yeast genetic screens helped to define the molecular basis of autophagy (Tsukada & Ohsumi, 1993; Harding *et al*, 1995). This approach led to the identification of autophagy‐related (ATG) genes, and whilst the exact number is debated, approximately 20 of these are “core” ATG genes, conserved across eukaryotes and encoding proteins essential for both non‐selective and selective autophagy (Tsukada & Ohsumi, 1993). Auxiliary ATG proteins enhance the autophagic process and/or participate in selective autophagy, though degradative autophagy in any case requires functional lysosomal proteins (Tanaka *et al*, 2000). One of the key molecular signatures of autophagy is ATG8 lipidation, a process whereby ATG8 is conjugated to phosphatidylethanolamine (PE) embedded in the emerging phagophore, thus enabling ATG8 to become an integral part of the autophagic membrane (Martens & Fracchiolla, 2020). ATG8 lipidation is a particularly important event because ATG8 facilitates several stages of autophagy including phagophore expansion, cargo recruitment, autophagosome transport and lysosomal fusion. In mammals, there are six ATG8 homologues, classified in the LC3 or GABARAP protein subfamilies.

**Figure 2:**
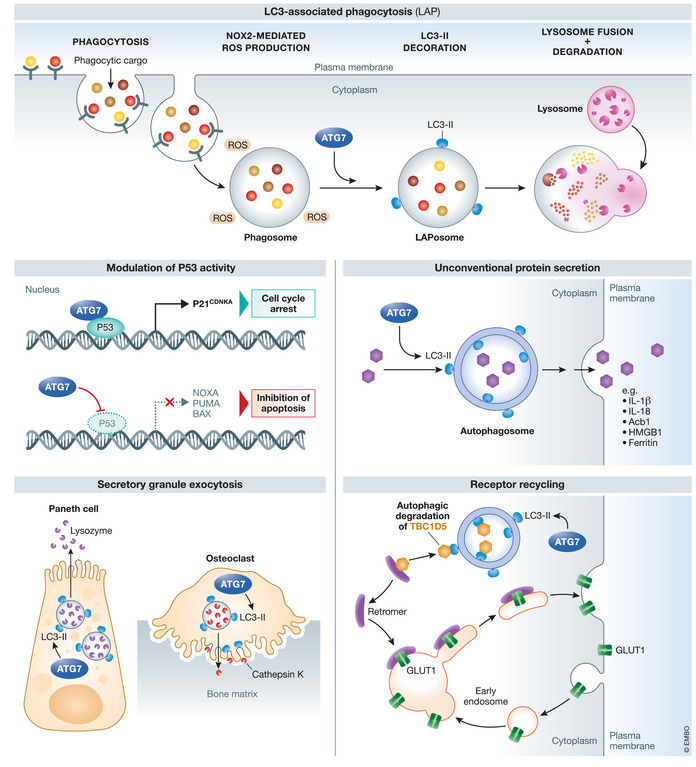
Overview of the autophagy‐related and autophagy‐independent biological functions of ATG7 Through its E1‐like enzymatic activity, ATG7 drives the conjugation of phosphatidylethanolamine (PE) to LC3‐I in a process termed “lipidation”, generating LC3‐II which is important for a number of physiological pathways beyond degradative autophagy. Independently of its E1‐like enzymatic activity, ATG7 modulates P53 activity, thus affecting cell cycle arrest and apoptosis via control of gene expression. ROS = reactive oxygen species. Adapted from Levine and Kroemer (2019).

A large body of evidence has demonstrated that ATG proteins contribute to a diverse range of biological processes that extend far beyond autophagy (Levine & Kroemer, 2019). ATG7 is one such multifaceted core ATG protein that drives the cardinal stages of classical degradative autophagy through ATG8 lipidation. ATG7 also makes pivotal contributions to innate immunity via LC3‐associated phagocytosis, unconventional protein secretion, receptor recycling, exocytosis of secretory granules and modulation of p53‐dependent cell cycle arrest and apoptosis (Mizushima & Levine, 2020; Mizushima, 2020) (Figs 1 and 2). This review will describe the role of ATG7 in these pathways, and the breakthrough genetic models that have led to our understanding of how ATG7 deficiency affects mammalian physiology. We will explore the association of impaired ATG7 activity with human pathologies including neurodegeneration, cancer and infection and pay particular attention to the recently identified recessive congenital disorder of autophagy caused by inherited ATG7 dysfunction leading to neurological manifestations (Collier *et al*, 2021). Recent breakthroughs in delineating the role of ATG7 in cell biology and human disease have important implications for the development of therapeutics that regulate autophagy. Whereas activation of autophagy provides an attractive therapeutic approach to treat human neuropathology where impaired autophagy is implicated, evidence has also emerged that autophagy inhibition can improve cancer treatment outcomes (Mizushima & Levine, 2020).

## Biological functions of ATG7

### Classical degradative autophagy

ATG7 impairment classically renders cells and tissues as “autophagy deficient”, and the study of autophagy has underpinned the majority of ATG7‐focused research (Komatsu *et al*, 2005; Komatsu *et al*, 2007a; Matsumoto *et al*, 2008; Collier *et al*, 2021). During autophagy, the phagophore membrane is enriched with phosphatidylethanolamine (PE), an abundant phospholipid that has been reported to positively regulate autophagic activity (Rockenfeller *et al*, 2015). PE is important because it acts as the anchor for recruitment of cytosolic ATG8 to the emerging phagophore membrane (Fig 1B). There are two mammalian ATG8 subfamilies encoding six homologues to yeast atg8 protein. The first, LC3, has three members, LC3A, LC3B and LC3C, and the second, GABARAP, represents the remaining three homologues, GABARAP, GABARAPL1 and GABARAPL2 (Lee & Lee, 2016). By convention, ATG8 refers to both LC3 and GABARAP subfamilies, and upon lipidation becomes “ATG8‐PE”. Whereas conjugated GABARAP is similarly referred to as “GABARAP‐PE”, the lipidated form of LC3 is termed “LC3‐II”. Lipidation of ATG8 remains an important marker for evaluating levels of autophagy in tissue and cells (Mizushima *et al*, 1998, 2010; Ichimura *et al*, 2000). In mammalian cells, levels of LC3 lipidation in particular are used to estimate autophagic flux via immunoblotting, yet this should not detract from the biological importance of GABARAP proteins.

ATG8 lipidation is a multistep process, driven by the E1‐like enzymatic activity of homodimeric ATG7 (Tanida *et al*, 1999, 2001; Komatsu *et al*, 2001) (Fig 1B). First, the protease ATG4 exposes the C‐terminus glycine residue of ATG8, generating form I (e.g. LC3‐I) (Kirisako *et al*, 1999). This form is then activated by ATG7 via adenylation, before it is transferred to ATG3 where it is conjugated to PE to generate form II (e.g. LC3‐II) (Tanida *et al*, 1999; Ichimura *et al*, 2000; Taherbhoy *et al*, 2011) which localises to both the inner (IAM) and outer autophagosomal membranes (OAM), and is subsequently degraded upon autolysosome formation (Kabeya *et al*, 2004). ATG7 is also involved in a second autophagy conjugation reaction that supports ATG8 lipidation. During this reaction, ATG12 is adenylated by ATG7 then transferred to ATG5 via E2‐like enzyme ATG10, generating ATG5‐ATG12 conjugates (Mizushima *et al*, 1998; Shintani *et al*, 1999; Tanida *et al*, 1999; Yamaguchi *et al*, 2012) which are restricted to the OAM and removed prior to sealing of the autophagosome (Koyama‐Honda *et al*, 2013). Although LC3‐II can be generated *in vitro* in the presence of ATG3, ATG7, LC3, ATP and liposomes containing PE, ATG5‐ATG12 forms a complex with ATG16L that promotes LC3 lipidation *in vivo* (Mizushima *et al*, 1999, 2003; Kuma *et al*, 2002; Hanada *et al*, 2007; Lystad *et al*, 2019). Evidence currently suggests that WIPI2 localises to PI3P‐rich regions of the phagophore membrane, recruiting the ATG5‐ATG12‐ATG16L1 complex that binds ATG3, which transfers activated ATG8 to membrane‐bound PE. Oxidation of ATG3 and ATG7 facilitates autophagy inhibition (Frudd *et al*, 2018).

Endogenous *ATG7* deletion prevents ATG8 lipidation, so *ATG7* knockout (KO) models are commonly used to study the biological significance of the autophagy conjugation systems. ATG8 has diverse roles in the autophagy pathway (Lee & Lee, 2016; Johansen & Lamark, 2020). First, mammalian LC3‐II is important for autophagosome maturation, with levels of lipidated LC3 correlating with the extent of autophagosome formation (Kabeya *et al*, 2004) and autophagic structures generated in the absence of ATG8 homologues are smaller (Nguyen *et al*, 2016). It was recently demonstrated that attachment of ATG8 to the phagophore membrane stimulates membrane deformation, leading to expansion of this structure and underpinning efficient autophagosome formation (Maruyama *et al*, 2021). Blockade of mammalian ATG8 lipidation through *ATG3* deletion caused delayed autophagosome maturation and a significant reduction in the success rate of autophagosome formation (Tsuboyama *et al*, 2016). In fact, a number of proteins involved in the early and late stages of autophagy have LIRs, emphasising the ability of ATG8 family proteins to coordinate multiple stages of the autophagic process (Martens & Fracchiolla, 2020) (Fig 1D). Beyond autophagic membrane expansion, ATG8 homologues facilitate transport of autophagosomes along microtubules via interactions with motor proteins via adaptor proteins and contribute to the binding of autophagosomes to lysosomes (Lorincz & Juhasz, 2020; Martens & Fracchiolla, 2020). LC3B can also be phosphorylated to regulate autophagosome transport (Nieto‐Torres *et al*, 2021). Moreover, loss of ATG7 impairs inner autophagosomal membrane (IAM) degradation after autophagosome–lysosome fusion (Tsuboyama *et al*, 2016). Consequently, autophagy is severely impaired by *ATG7* deletion as evidenced in yeast, mouse and humans (Tanida *et al*, 1999; Komatsu *et al*, 2005; Luhr *et al*, 2018).

One of the most widely studied aspects of ATG8 lipidation is its requirement for selective autophagy which is currently defined by the recognition, sequestration and elimination of specific cytoplasmic cargo. This selectivity is achieved through the interaction of cargo with ATG8 via LIR motifs within specific receptors that act as “eat‐me” signals for damaged or excess cellular components (Johansen & Lamark, 2020) (Fig 1C). Cargo types include organelles such as mitochondria (termed *mitophagy*), endoplasmic reticulum (*ER‐phagy* or *reticulophagy*) and peroxisomes (*pexophagy*), proteins and protein aggregates (*aggrephagy*), and intracellular pathogens (*xenophagy*). Consequently, selective autophagy is an important homeostatic mechanism, preventing the accumulation of dysfunctional organelles, cytotoxic aggregates and providing innate immune support, as well as a developmental tool facilitating the removal of mitochondria from maturing reticulocytes, cardiomyocytes, kidney cells and ocular tissues, for example (Sandoval *et al*, 2008; Mortensen *et al*, 2010; McWilliams *et al*, 2016, 2019; Esteban‐Martinez *et al*, 2017). LIR motifs can be regulated through masking and activating/inhibitory phosphorylation to prevent promiscuous cargo sequestration under conditions where the autophagic degradation of that substrate or organelle has not been stimulated (Chen *et al*, 2014, 2016; Lv *et al*, 2017; Wei *et al*, 2017). Autophagic sequestration of mitochondrial proteins can also be regulated by acetylation (Webster *et al*, 2013). Although the fundamental mechanisms driving cargo selection are shared between ATG8 homologues, there is evidence of homologue‐specific autophagy adaptor proteins (Wirth *et al*, 2019).

### Autophagy‐related functions

Autophagy‐related functions of ATG7 involve membrane trafficking events that are dependent on LC3 lipidation. Consequently, these processes require the activity of other core ATG proteins that drive lipidation of ATG8 homologues, including ATG3, ATG5 and ATG12—members of the autophagy conjugation system (Mizushima, 2020) (Fig 1). This includes the innate immune process LC3‐associated phagocytosis (LAP) (Heckmann & Green, 2019; Inomata *et al*, 2020) (Fig 2). During LAP, extracellular pathogens, dead/dying cells and other extracellular substrates are recognised by cell surface receptors then endocytosed, generating an intracellular single‐membrane structure called the phagosome. Then, LC3‐II, generated in an ATG7‐dependent manner, is recruited to the phagosome in a process dependent on NOX2‐derived ROS (Sanjuan *et al*, 2007; Martinez *et al*, 2015). This structure, the LAPosome, is now decorated with LC3‐II and able to fuse with lysosomes for elimination (Sanjuan *et al*, 2007). A related process, termed entosis, is also dependent on LC3 lipidation. During entosis, viable cells are engulfed by epithelial cells. This process is regulated by the cell being engulfed, after which this cell undergoes non‐apoptotic cell death driven by autophagosomes and lysosomes of the host cell (a process termed “non‐cell autonomous autophagy”). When autophagy in the host cell is inhibited, the engulfed cell largely undergoes apoptosis (Florey *et al*, 2011), whereas others have been observed to divide inside the host cell, or escape into culture (Overholtzer *et al*, 2007). Tumour cells can also undergo entosis (Overholtzer *et al*, 2007; Fais & Overholtzer, 2018).

The activities of ATG7 can also be non‐degradative. For example, LAP also facilitates Toll‐like receptor 9 (TLR9) trafficking and converges with the classical autophagy pathway to regulate IFN‐alpha production (Henault *et al*, 2012). ATG7‐mediated LC3 lipidation is also required for the exocytic release of cathepsin K by osteoblasts (DeSelm *et al*, 2011), and LC3‐positive lysozyme‐containing granules are released by Paneth cells upon infection (Bel *et al*, 2017). Related to this, autophagosomes facilitate the unconventional secretion of proteins including IL1B and ferritin in response to lysosomal damage (Kimura *et al*, 2017), and loss of ATG7 causes accumulation of mucin granules in Goblet cells (Patel *et al*, 2013). Autophagosomes are also able to sequester TBC1D5, thus freeing the retromer complex to mediate the return shuttling of GLUT1 transporters to the plasma membrane from endosomes (Roy *et al*, 2017). As part of the autophagic machinery, ATG7 is also involved in regulating the switch between apoptosis and necrosis (Goodall *et al*, 2016). This important study demonstrated that the necrosome (a protein complex that leads to rapid plasma membrane rupture and inflammation) can assemble on autophagosomes at selective autophagy receptor p62 sites and that loss of p62 can switch cell death mechanisms towards apoptosis.

### Autophagy‐independent functions

ATG7 also participates in cellular functions that are independent of its E1‐like enzymatic activity. Consequently, these functions do not require the other ATG machinery required for autophagy‐associated signalling. A number of autophagy‐independent functions of ATG7 have been described, with two involving modulation of p53 activity. Upon starvation, Atg7 has been reported to interact with p53 to inhibit the expression of pro‐apoptotic genes *Noxa*, *Puma* and *Bax*. Accordingly, *Atg7*‐null mouse embryonic fibroblasts demonstrated augmented DNA damage under basal conditions. The proliferation of *Atg7*‐null cells proceeded at a far greater rate than control cells due to diminished p53‐mediated *p21* expression, which usually promotes cell cycle arrest (Lee *et al*, 2012). In another study, Atg7 was also shown to repress the pro‐apoptotic properties of caspase 9 (Han *et al*, 2014). An isoform of ATG7 that lacks E1‐like enzymatic activity has also been discovered, and this variant cannot lipidate ATG8 homologues (Ogmundsdottir *et al*, 2018). The biological function of this intriguing isoform is unknown, but it may negatively regulate autophagy by potentially disrupting the formation of functional ATG7 homodimers.

## Mouse models of *Atg7* deficiency

A prodigious body of evidence, largely attained through studying mouse genetic models, has demonstrated that faithful ATG7 function is essential for the development, maintenance and adaptation of mammalian tissues (Xiong, 2015). Embryonic *Atg7* deletion in mice results in perinatal lethality, and the subsequent characterisation of conditional Atg7 deficiency in mice has illuminated the contribution of ATG7 to mammalian physiology (Table 1). It is notable that manipulation of other core *Atg* genes causes similar phenotypes to those observed in *Atg7* KO models, supporting the role of autophagy in these discoveries. Here, we discuss these key genetic mouse models, exploring the phenotypic and cellular consequences of endogenous inhibition of mammalian ATG7.

**Table 1:** Overview of Atg7‐deficient mouse models.

Knockout	Phenotypes	References
Whole body (embryonic)	Perinatal lethal	Komatsu *et al* (2005)
Whole body (adult)	Neurodegeneration Susceptibility to infection	Karsli‐Uzunbas *et al* (2014)
Central nervous system	Neurodegeneration Ataxia Behavioural abnormalities	Kim *et al* (2017), Komatsu *et al* (2006), Komatsu *et al* (2007b)
Liver	Liver enlargement Multiple adenomas	Komatsu *et al* (2007a), Komatsu *et al* (2005)
Skeletal muscle	Loss of muscle mass and strength Impaired exercise adaptation	Lo Verso *et al* (2014), Masiero *et al* (2009)
Circulatory system	Diabetic cardiomyopathy Susceptible to ischaemic injury	Saito *et al* (2019), Tong *et al* (2019)
Pancreas	Premature death Hyperglycaemia Insulin deficiency Endotoxin‐induced chronic pancreatitis	Xia *et al* (2020), Zhou *et al* (2017)
Adipose	Loss of white adipose tissue mass Insulin sensitivity	Singh *et al* (2009b), Zhang *et al* (2009b)
Haematopoiesis	Severe anaemia Inability to reconstitute irradiated mice	Mortensen *et al* (2010), Mortensen *et al* (2011)
Bone	Reduced bone mass Short tibia and femur	Li *et al* (2018)
Intestine	Susceptible to infection	Inoue *et al* (2012b)
Ear	Early‐onset hearing loss	Zhou *et al* (2020)
Eye	Retinal degeneration	Zhang *et al* (2017)

### Systemic or whole‐body Atg7 deletion

Similarly to the majority of other core *Atg* genes, systemic knockout of *Atg7* in mice causes death within 24 h after birth (Komatsu *et al*, 2005). The neonatal lethal phenotype is recapitulated across other core *Atg* genes, including *Atg5* (Kuma *et al*, 2004). It was then demonstrated that neural reconstitution of Atg5 activity in *Atg5*‐null mice prevents neonatal death (mice die between 8 weeks and 8 months after birth), revealing that neural dysfunction is the primary cause of perinatal death in whole‐body knockout animals. This is possibly due to a suckling defect (Yoshii *et al*, 2016), although *Atg7*‐ and *Atg5*‐null mice died before wild‐type mice, even under non‐suckling conditions (Kuma *et al*, 2004; Komatsu *et al*, 2005). Conditional models such as tamoxifen‐inducible whole‐body *Atg7* deletion in adult mice impaired glucose metabolism, causing death 2–3 months post‐knockout due to neurodegeneration, and fasting these mice caused fatal hypoglycaemia (low blood glucose levels) and cachexia (muscle wasting) (Karsli‐Uzunbas *et al*, 2014). Amino acid levels were also diminished in *Atg7* KO mice (Komatsu *et al*, 2005). A combination of defective LAP and autophagy may underlie the susceptibility of inducible adult *Atg7* KO mice to *Streptococcus* infection (Karsli‐Uzunbas *et al*, 2014). Adult mice with concurrent *Atg7* and *p53* deletion have similar lifespan to *p53* KO mice, and the double KO mice died from neurodegeneration without the tumour development that was observed in *p53* KO mice (Yang *et al*, 2020). The double KO mice were more resistant to fasting, and liver and brain injury was decreased due to protection against apoptosis and DNA damage (Yang *et al*, 2020).

### Central nervous system

Perhaps the most striking physiological effects of Atg7 deficiency manifest in the central nervous system, where conditional *Atg7* KO caused neurodegeneration resulting in premature death (Komatsu *et al*, 2006). Mice also displayed an ataxic phenotype caused by selective vulnerability of cerebellar Purkinje neurons to Atg7 deficiency (Komatsu *et al*, 2006; Komatsu *et al*, 2007b) and behavioural abnormalities that are recapitulated in mice with myeloid‐specific *Atg7* deletion via impaired microglial synaptic refinement (Kim *et al*, 2017). Other regions in the brain affected by *Atg7* deletion include the hypothalamus through impaired lipolysis and glucose homeostasis (Coupe *et al*, 2012; Kaushik *et al*, 2012), the forebrain via phosphorylated tau accumulation (Inoue *et al*, 2012b; Nilsson *et al*, 2013), and midbrain dopaminergic neurons through dysregulated presynaptic neurotransmission (Hernandez *et al*, 2012). Mice with neural stem cell‐specific *Atg7* KO were resistant to stress‐induced cognitive deficits due to impaired cell death (Jung *et al*, 2020). It was also found that p62‐positive neuronal inclusions hallmark defective autophagy in Atg7‐deficient brain models (Komatsu *et al*, 2007a). This discovery cannot be overstated, as it provided a direct link between autophagy and the accumulation of proteinaceous inclusions that are characteristic of human neurodegenerative pathology.

### Liver

Atg7‐deficient mice demonstrated an impaired fasting response and enlarged livers, as well as the accumulation of abnormal organelles and p62 and ubiquitin‐positive inclusions (Komatsu *et al*, 2005, 2007a). Impaired lipid metabolism, leading to increased cholesterol and triglyceride content, also contributes to liver injury (Singh *et al*, 2009a). Initially, it was thought that autophagy is required for progression of liver tumours to malignancy, as *Atg7* KO caused adenoma formation but no cancerous tumours were detected (Takamura *et al*, 2011). However, a subsequent study reported that liver‐specific *Atg7* deletion causes hepatocellular carcinoma at 12 months (Lee, Noon, *et al*, 2018), with the authors commenting that this may be because of differences in genetic background and microbial environment.

Accumulation of p62 in liver appears to play an important role in pathology. P62 primarily acts as an adaptor protein in autophagy, but is also involved in the non‐canonical regulation of the activity of oxidative stress‐inducible transcription factor NRF2 (Sanchez‐Martin & Komatsu, 2018). P62 competitively interacts with KEAP1, which usually binds NRF2 to prevent its nuclear translocation. Thus, p62 accumulation enhances the release of NRF2, initiating an antioxidant transcriptional response (Inami *et al*, 2011). Although p62 accumulates in both *Atg7* KO liver and brain, only hepatic phenotypes are rescued by concurrent *p62/SQSTM1* or *NFE2L2* (encoding NRF2) deletion, defining tissue‐specific mechanisms underlying *Atg7*‐related pathology (Komatsu *et al*, 2007a, 2010; Takamura *et al*, 2011). In support, antioxidant gene expression is upregulated in *Atg7* KO liver but not brain (Komatsu *et al*, 2006; Matsumoto *et al*, 2008). It has also been shown that concurrent deletion of *Atg7* and *Yap*, which like p62 is degraded via autophagy, prevents hepatomegaly and tumorigenesis (Lee *et al*, 2018b). The p62‐KEAP1‐NRF2 axis is maintained in these double KO livers, suggesting that even within individual tissues multiple mechanisms may contribute to pathogenesis.

### Skeletal muscle

Atg7 is required in skeletal muscle for development, basal homeostasis and adaptation. Loss of Atg7 in embryonic or adult skeletal muscle caused loss of muscle mass and strength, with abnormal mitochondria, swollen sarcoplasmic reticulum, internalised nuclei and vacuoles notable (Masiero *et al*, 2009). Faithful Atg7 function also protected mice against exercise‐mediated mitochondrial dysfunction (Lo Verso *et al*, 2014). Conversely, skeletal muscle‐specific *Atg7* deletion is protective against diet‐induced obesity and diabetic phenotypes. Basal mitochondrial dysfunction in Atg7‐deficient skeletal muscle activates the integrated stress response via *Atf4* which promotes *Fgf21* expression, stimulating fatty acid oxidation (Kim *et al*, 2013).

### Circulatory system

Atg7 appears to protect against diabetic cardiomyopathy. High‐fat diet feeding of mice with cardiac‐specific *Atg7* deletion exacerbated lipid accumulation and diastolic dysfunction, leading to systolic dysfunction (Tong *et al*, 2019). However, Atg7 is dispensable for protection against physiological energetic stress via starvation or ischaemia (Saito *et al*, 2019). Loss of Atg7 in vascular smooth muscle accentuated basal Ca^2+^ concentrations and heightened sensitivity to depolarisation (Michiels *et al*, 2015). Cultured Atg7‐deficient smooth muscle cells showed diminished survival and proliferation although they demonstrated increased resistance to oxidative stress‐induced cell death, reportedly due to increased NRF2 nuclear translocation and antioxidant gene expression (Grootaert *et al*, 2015; Osonoi *et al*, 2018). Endothelial‐specific *Atg7* deletion did not affect vessel architecture or capillary density but did impair adrenaline‐mediated von Willebrand factor release leading to extended bleeding times (Torisu *et al*, 2013). Atg7‐deficient endothelial cells also exhibited diminished fatty acid storage (Altamimi *et al*, 2019) and augmented endothelial to mesenchymal transition (Singh *et al*, 2015).

### Pancreas

In the pancreas, loss of Atg7 causes premature death due to declining exocrine and endocrine function (Zhou *et al*, 2017). Atg7 deletion also appears to increase susceptibility to endotoxin‐induced chronic pancreatitis (Xia *et al*, 2020). Atg7 maintains islet architecture, glucose tolerance and serum insulin levels and is important for islet homeostasis and compensatory pancreatic responses to high‐fat diet (Ebato *et al*, 2008; Jung *et al*, 2008; Himuro *et al*, 2019). Atg7‐deficient beta cells accumulate dysfunctional organelles, and impaired proliferation and increased apoptosis cause hyperglycaemia and insulin deficiency (Jung *et al*, 2008).

### Adipose tissue

Mice with adipose‐specific *Atg7* deletion demonstrated impaired metabolic homeostasis. Loss of *Atg7* led to a reduction of 80% of white adipose tissue mass, which acquired brown adipose tissue features including accumulation of mitochondria (Singh *et al*, 2009b; Zhang *et al*, 2009b). Tissue demonstrated increased beta‐oxidation and diminished hormone‐induced lipolysis, supporting the finding of altered lipid metabolism in other Atg7‐deficient models (Singh *et al*, 2009a) and leading to attenuation of basal fatty acid plasma concentration and increased insulin sensitivity (Singh *et al*, 2009b; Zhang *et al*, 2009b). Loss of *Atg7* from brown adipose tissue also elevated mitochondrial content and insulin sensitivity (Kim *et al*, 2019).

### Haematopoiesis

The role of Atg7 in the haematopoietic system has been extensively investigated. Atg7 is required for erythroid differentiation by contributing to mitochondrial clearance during erythrocyte maturation (Zhang *et al*, 2009a; Cao *et al*, 2016). Although removal of the endoplasmic reticulum and ribosomes was unaffected by *Atg7* deletion, subsequent accumulation of damaged mitochondria caused severe anaemia (Mortensen *et al*, 2010). Atg7 deficiency also caused apoptosis induced by mitochondrial damage in mature T lymphocytes leading to lymphopenia (Mortensen *et al*, 2010). Another study reported that T‐cell‐specific *Atg7* deletion impairs IL‐2 and IFN‐y production and impairs stimulated proliferation but does not induce apoptosis (Hubbard *et al*, 2010). ER content and calcium stores were increased in Atg7‐deficient T cells, impairing cellular calcium influx (Jia *et al*, 2011). Haematopoietic stem cell‐specific *Atg7* KO caused death within weeks, with haematopoietic stem and progenitor cells demonstrating increased ROS, mitochondrial mass, proliferation and DNA damage (Mortensen *et al*, 2011). Lymphoid and myeloid progenitor production was impaired, and Atg7‐deficient stem cells were unable to reconstitute the haematopoietic system of irradiated mice (Mortensen *et al*, 2011). Myeloid‐specific *Atg7* KO did not affect metabolism but did increase inflammasome activation, ROS production and IL1B release after palmitic acid and lipopolysaccharide treatment (Lee *et al*, 2016). Atg7 is required for normal monocyte differentiation and acquisition of phagocytic function by macrophages (Jacquel *et al*, 2012). In B cells, *Atg7* deletion caused selective loss of B1a B cells through impaired self‐renewal. Atg7‐deficient B1a B cells accumulated dysfunctional mitochondria and exhibited diminished expression of metabolic genes, which was not as severe in the less autophagy‐dependent B2 B cells (Clarke *et al*, 2018).

### Bone

Chondrocyte‐specific *Atg7* deletion induced ER type II procollagen storage, driving the aberrant formation of the Col2 fibrillary network in the extra cellular matrix, despite normal Col2 levels (Cinque *et al*, 2015). This led to diminished tibial and femoral lengths in Atg7‐deficient mice. Interestingly, pharmacological activation of autophagy rescued phenotypes in *Fgf18*‐ or *Fgfr4*‐deficient mice, suggesting that autophagy is activated by FGF signalling in bone (Cinque *et al*, 2015). Different models have suggested that chondrocyte‐specific *Atg7* deletion induces apoptosis and decreases chondrocyte proliferation (Vuppalapati *et al*, 2015; Kang *et al*, 2017). *CHOP* deletion partially reversed impaired chondrocyte dysfunction, implicating the PERK‐ATF4‐CHOP axis and thus supporting the role of ER‐related stress in pathophysiology (Cinque *et al*, 2015; Kang *et al*, 2017). Loss of *Atg7* from osteoblasts caused a decrease in bone mass during development and adulthood due to diminished osteoblast formation and matrix mineralisation, as well as increased numbers of osteoclasts (Li *et al*, 2018). Like other Atg7‐deficient bone models, ER stress was upregulated. Relief from ER stress using phenylbutyric acid remedied bone‐related phenotypes in several Atg7‐deficient models, placing endoplasmic reticulum dysfunction at the heart of pathology (Kang *et al*, 2017; Li *et al*, 2018).

### Intestine

Deletion of *Atg7* from the intestinal epithelium disrupts Paneth cell morphology and affects Paneth cell granule size, morphology and number (Cadwell *et al*, 2009; Wittkopf *et al*, 2012). Although histological analysis of the small intestine revealed no changes after *ATG7* deletion, mice demonstrated increased expression of pro‐inflammatory genes and accentuated endotoxin‐induced inflammatory responses due to increased NF‐kB activity (Cadwell *et al*, 2009; Fujishima *et al*, 2011; Inoue *et al*, 2012a). Atg7‐deficient mice were also susceptible to *Citrobacter rodentium* infection and infected mice displayed increased disease burden, possibly due to impaired LAP (Inoue *et al*, 2012a). Loss of *Atg7* from intestinal antigen presenting cells elevated mitochondrial ROS production and T_H_17 inflammation (Ravindran *et al*, 2016). Related to this, *Atg7* deletion from intestinal stem cells increased oxidative stress, altered gut–microbiota interactions, and impaired DNA repair, contributing to the induction of p53‐mediated apoptosis (Trentesaux *et al*, 2020). Consistent with liver and brain models, concurrent p53 deletion reduced cell death (Trentesaux *et al*, 2020; Yang *et al*, 2020).

### Eye and ear

A number of other important Atg7‐deficient mouse models have been studied. Outer hair cell *Atg7* deletion resulted in accumulation of dysfunctional mitochondria, causing profound early‐onset hearing loss (Zhou *et al*, 2020), and Atg7 deficiency in retinal pigmented epithelia predisposed mice to retinal degeneration (Zhang *et al*, 2017). Interestingly, organelle degradation is normal in the developing lens of autophagy‐deficient mice and instead depends upon PLAAT (phospholipase A/acyltransferase) phospholipases (Morishita *et al*, 2021).

## Non‐mammalian models of atg7 deficiency

Studies using other models of Atg7 deficiency support numerous findings in mouse models. *Atg7* KO *Drosophila melanogaster* (fruit flies) have reduced lifespan, are sensitive to nutrient and oxidative stress and demonstrate an ataxic‐like phenotype associated with degenerating neurons (Juhasz *et al*, 2007). In *Caenorhabditis elegans* (nematode roundworm), *atg7* is required for longevity in the dietary restriction *eat‐2* mutant (Jia & Levine, 2007), but not in the insulin‐resistant *daf‐2* longevity model (Hashimoto *et al*, 2009). Morpholino *atg7* knockdown in *Danio rerio* (zebrafish) causes aberrant cardiac morphogenesis, increasing the number of dead cells and attenuating organism survival (Lee *et al*, 2014).

## ATG7 in human disease

**Figure 3:**
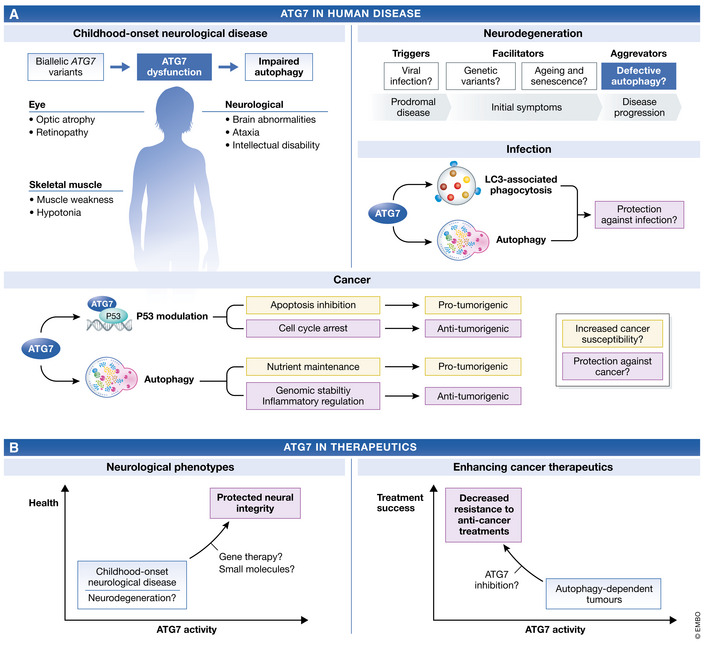
ATG7 in human disease and therapeutics (A) Patients harbouring biallelic *ATG7* variants display childhood‐onset disease, causing neurological, muscular and ocular dysfunction through impaired autophagy. In contrast, there is no direct link between ATG7 and adult‐onset neurodegeneration, infection or cancer, although recent developments in our understanding of these diseases and ATG7 function have implicated aberrant ATG7 activity in their aetiology. (B) This has implications for therapeutic approaches. Whereas neurological phenotypes may be remedied by induction of ATG7 activity, inhibition of ATG7 may help increase the efficacy of anti‐cancer treatments.

Dysfunctional autophagy has been predicted to underpin a number of human diseases (Fig 3). Given that autophagy‐deficient mice manifest profound neurodegenerative phenotypes, it has been proposed that impaired autophagy in humans may underlie neurodegenerative conditions including Alzheimer’s disease (AD), Parkinson’s disease (PD) and amyotrophic lateral sclerosis (ALS) (Fujikake *et al*, 2018). The relationship between autophagy and cancer is particularly complex, and aberrant autophagic activity is proposed to play a context‐specific role in human carcinogenesis (Long & McWilliams, 2020). Involvement of ATG7 in cancer is further complicated by its autophagy‐independent ability to modulate cell cycle arrest and apoptosis mediated by the tumour suppressor p53 (Lee *et al*, 2012). The role of ATG7 in LC3‐associated phagocytosis suggests that ATG7 deficiency may lead to increased susceptibility to infection, supported by the predisposition of Atg7‐deficient mice to infection (Inoue *et al*, 2012a; Karsli‐Uzunbas *et al*, 2014). Until we recently discovered a series of patients harbouring pathogenic, biallelic *ATG7* variants, there was no direct link between ATG7 and human disease. This section will describe the clinical nature of these patients and assess the contribution of ATG7 dysfunction to complex human disorders.

### Childhood‐onset neurological disease

Congenital disorders of autophagy are an emerging group of inborn errors of metabolism, primarily affecting the central nervous system with common involvement of the cerebellum and corpus callosum (Teinert *et al*, 2020). Although the genetic aetiology underpinning these conditions is expanding, congenital autophagy disorders remain incredibly rare. In fact, deleterious variants affecting only five core autophagy genes have been reported, including *ATG5*, *WIPI2*, *WDR45*, *WDR45b* and *ATG7* following the very recent description of twelve patients from five, unrelated families harbouring deleterious, biallelic *ATG7* variants (Haack *et al*, 2012; Saitsu *et al*, 2013; Kim *et al*, 2016; Suleiman *et al*, 2018; Jelani *et al*, 2019; Collier *et al*, 2021).

Patients with recessive *ATG7* variants are primarily affected by neurological abnormalities including mild‐to‐severe intellectual disability, ataxia and tremor (Collier *et al*, 2021). Brain magnetic resonance imaging (MRI) revealed cerebellar hypoplasia and a thin posterior corpus callosum in all patients who had been assessed, highlighting the selective vulnerability of these regions to ATG7 deficiency. Assessment of patient skeletal muscle with undetectable ATG7 protein revealed myopathic changes, including subsarcolemmal accumulation of p62 and evidence of inflammation. Related to this, the patient cohort also displayed neuromuscular abnormalities including loss of muscle mass and strength. Ocular dysfunction, predominantly optic atrophy, is commonly displayed by patients, and there was evidence of endocrine dysfunction and behavioural abnormalities. In more severe cases, patients have seizures and are wheelchair bound due to spastic paraplegia, and one patient died during childhood.

Biochemical profiling of patient fibroblasts revealed severely diminished ATG7 protein levels. Mechanistically, this manifested with impairments in autophagic flux, evidenced by both diminished LC3‐II accumulation and decreased cargo sequestration activity. Complementation of *atg7* KO *Saccharomyces cerevisiae* and *ATG7* KO mouse embryonic fibroblasts by missense *Atg7* variants, which were predicted to interfere with ATG7 homodimerisation, failed to rescue autophagy to wild‐type levels, thus consolidating variant pathogenicity. Remarkably, two siblings studied survived into adulthood despite undetectable ATG7 causing a near absence of autophagic flux and severely attenuated long‐lived protein degradation, supporting the idea that human life is compatible, in exceptional circumstances, with loss of a nonredundant core autophagy protein. Moreover, *ATG7* patients with dramatic reduction in autophagic activity are now approaching population life expectancy.

These findings consolidated the importance of autophagy in human health, providing a direct link between dysfunctional autophagy and neurological disease in multiple families. A key question that has arisen from this study is as follows: How do humans compensate for loss of classical degradative autophagy? Based on logical assumptions derived from studies on mouse *Atg5/Atg7*, it was thought that loss of *ATG7*—or any of the nonredundant core ATG genes—would not be compatible with human survival. Studies using autophagy‐dependent cancer cell lines have demonstrated that they can adapt to autophagy inhibition through increasing mitochondrial‐derived vesicle production and inducing NRF2 signalling (Towers *et al*, 2019, 2021). Moreover, an ATG7/ATG5‐independent autophagy pathway driven by RAB9 has also been described (Nishida *et al*, 2009). This pathway was also suggested to contribute to mitophagy (Hirota *et al*, 2015; Saito *et al*, 2019), and the delineation of other molecular signatures of this pathway will enhance our understanding of its role in human intracellular degradation. It has also been reported that FIP200 clustering enables selective autophagy in the absence of LC3 lipidation (Ohnstad *et al*, 2020). Continued clinical assessment of these patients will provide further insight into the role of ATG7 in human homeostasis and disease. The future identification of additional individuals with inherited *ATG7* impairments will reveal the spectrum of clinical phenotypes associated with ATG7 dysfunction. This is important because the patients described so far do not demonstrate predictable clinical outcomes. This is exemplified by the discrepancy between clinical and biochemical phenotypes whereby patients with the most severe clinical presentations demonstrated the mildest biochemical impairment of autophagy. These approaches will also help to ascertain whether ATG7‐deficient individuals are at altered risk of diseases such as neurodegeneration, cancer and infection compared with the general population, improving our understanding of complex human disorders.

### Adult neurodegeneration

Neurodegeneration is characteristic of autophagy‐deficient mouse models, yet the relationship between defective autophagy and human neurodegeneration has been more challenging to reconcile (Suomi & McWilliams, 2019). Strong evidence supporting the pathological involvement of autophagy includes the presence of large p62/SQSTM1‐ and ubiquitin‐positive inclusions, hallmarks of autophagy deficiency, in AD, PD and ALS brain tissues (Kuusisto *et al*, 2001; Mizuno *et al*, 2006). In support, these structures often contain autophagy cargo including TDP‐43, hyperphosphorylated tau, SOD1 and alpha‐synuclein (Menzies *et al*, 2017). The discovery of patients harbouring recessive *ATG7* variants has resolved a long‐suspected convergence of neuropathology between humans and model organisms with defective autophagy (Collier *et al*, 2021). Longitudinal assessment of patients harbouring these pathogenic variants will inform whether and how their neuropathological status changes over time. It is notable that one patient (71 years old) has developed late‐onset dementia decline, yet further studies are warranted before this can be attributed to ATG7 deficiency.

Despite this, mutations in several genes that participate at multiple steps of autophagy have been implicated in familial neurodegeneration (Menzies *et al*, 2017; Suomi & McWilliams, 2019). However, there is evidence that the contribution of selective autophagy proteins to neurodegeneration may extend beyond their involvement in selective mechanisms alone. For example, dysfunction of the mitophagy‐associated protein PINK1/PARK6 is implicated in PD pathogenesis, yet mouse, fly and even non‐human primate models have demonstrated that basal mitophagy is unaffected by endogenous inactivation of the Pink1‐Parkin pathway (Lee *et al*, 2018a; Marcassa *et al*, 2018; McWilliams *et al*, 2018a,b; Yamada *et al*, 2018; Yang *et al*, 2019; Wrighton *et al*, 2021). Rather, there is evidence that divergent mechanisms of impaired immune regulation contribute to acute PD‐like phenotypes. An important example is that intestinal infections trigger the manifestation of authentic PD‐like characteristics in *Pink1* KO mice, with locomotor dysfunction rescued by L‐DOPA treatment (Matheoud *et al*, 2019).

These data support an emerging theory proposing that a combination of triggers, facilitators and aggravators may affect Parkinson’s disease risk (Johnson *et al*, 2019). In short, triggers (e.g. viral infection) initiate disease but facilitators (e.g. genetic variant) may drive the spread of pathology, before the progressive loss of health due to aggravators (e.g. impaired autophagy). Given the often sporadic nature of other complex neurodegenerative conditions, it is reasonable to postulate that this may be true for these disorders, too, though the underlying mechanisms may differ between them. Although no direct links between ATG7 dysfunction and neurodegeneration have been demonstrated, polymorphisms within the *ATG7* promoter region have been suggested to contribute to sporadic Parkinson’s disease, yet their impacts on promoter activity were modest (Chen *et al*, 2013). Recently, it was reported that ATG7 protein levels are reduced in post‐mortem frontotemporal lobe brain tissue from ALS patients compared with controls, though causality in human subjects has not yet been proven (Donde *et al*, 2020). *ATG7* variants may also provide disease‐modifying effects, as it has been reported that the p.Val471Arg (NP_ 006386.1) polymorphism is associated with earlier onset (by 4–6 years) of Huntington’s disease (Metzger *et al*, 2010, 2013). These studies support the future consideration of ATG7 dysfunction as an aggravator of neurodegeneration, contributing to disease progression, rather than as a trigger of neurodegeneration. However, further research will be undoubtedly required to define the role of ATG7 in human neurodegeneration.

### Cancer

In human cancers, autophagy was first thought to be an anti‐tumorigenic process with *ATG6/BECN1* haploinsufficiency detected in approximately 45 to 70% of breast, ovarian and prostate cancers (White, 2015). Yet, other core autophagy genes, including *ATG7*, are rarely mutated in human cancers. It has instead become evident that the relationship between cancer and autophagy is undoubtedly complex, underpinned by the context‐specific interactions of pro‐ and anti‐tumour properties of autophagy (Long & McWilliams, 2020). Whereas autophagy protects against tumour formation by promoting genomic stability and inhibition of pro‐oncogenic inflammatory signalling, it also helps meet the accentuated metabolic demands of tumour microenvironments that result from increased proliferation (Zhong *et al*, 2016; Hewitt & Korolchuk, 2017). For example, *RAS* transformed cancers increase autophagic flux, a mechanism that may also be important for invasion and metastasis (Guo *et al*, 2011; Lock *et al*, 2011, 2014; Yang *et al*, 2011).

Because ATG7 promotes autophagy as well as cell cycle arrest mediated by tumour suppressor gene P53 (Lee *et al*, 2012), there is a logical expectation that altered ATG7 activity may underlie some cancers. Liver‐specific *Atg7* deletion predisposes mice to liver tumorigenesis, although these tumours were not reported to become malignant (Takamura *et al*, 2011). However, a subsequent study reported that *Atg7*‐null mice develop hepatocellular carcinoma (Lee *et al*, 2018b). Together, these data support a context‐specific role of *ATG*7 in carcinogenesis (perhaps driven by genetic and environmental cues including microbial exposure) and highlight that the relationship between autophagy and tumour formation and progression are even more complex that initially thought. In fact, cell non‐autonomous mechanisms are also an important consideration (Mizushima & Levine, 2020) and it was recently shown that tumour growth is supported by autophagy via circulating arginine (Poillet‐Perez *et al*, 2018). Elegant research using a *Drosophila melanogaster* malignant tumour model also supports the role of the non‐cell autonomous autophagy which was shown to be induced in the tumour microenvironment and in distal tissues (Katheder *et al*, 2017). In this model, early‐stage tumour growth and invasion was shown to be dependent on local tumour microenvironment autophagy. Studies have also been undertaken using murine cancer models. Conditional inactivation of *Atg7* inhibits intestinal pre‐cancerous lesion formation in mice with monoallelic deletion of tumour suppressor gene *Apc* (Levy *et al*, 2015) and prevents the growth of Braf^V600E^‐driven melanoma and lung tumours (Strohecker *et al*, 2013; Xie *et al*, 2015). However, autophagy inhibition drives the accumulation of pre‐malignant pancreatic lesions in mice harbouring an activated oncogenic *Kras* allele upon p53 inactivation (Rosenfeldt *et al*, 2013), whereas p53 deletion‐driven tumour formation (in the absence of Kras activation) is protected against by autophagy inhibition (Yang *et al*, 2020). Altogether, these studies demonstrated that the role of autophagy may be intrinsically linked to the status of oncogenes and tumour suppressors, as well as the metabolic microenvironment.

In humans, the link between ATG7 dysfunction and cancer formation is only starting to emerge. Recently, familial cholangiocarcinoma (an aggressive cancer of the bile duct) has been associated with *ATG7* mutations (preprint: Greer *et al*, 2019). In this study, a number of individuals harbouring inherited monoallelic *ATG7* variants (interestingly including the p.Arg659* (NP_ 006386.1) variant identified in Family 1 (Collier *et al*, 2021)) were discovered having developed cholangiocarcinoma. Tumour analysis revealed somatic loss of ATG7 affecting several family members, providing a strong link between cancer formation and ATG7 dysfunction. This discovery is particularly interesting given the prominent liver phenotypes, including tumour formation, observed in Atg7‐null mice (Takamura *et al*, 2011; Lee *et al*, 2018b). In contrast, there is currently no evidence for increased cancer susceptibility among patients harbouring recessive *ATG7* variants, nor in their family members with monoallelic *ATG7* variants, including those harbouring the p.Arg659* mutation. Among the patients with biallelic *ATG7* variants, a 71‐year‐old patient has developed an acoustic neuroma—a benign brain tumour, but longitudinal studies in other patients will provide further insight into whether this is related to ATG7 deficiency. Elsewhere, *ATG7* polymorphisms associated with protective or pro‐carcinogenic properties have been reported (Yu *et al*, 2018; Wang *et al*, 2019b), and elevation of *ATG7* expression is associated with some bladder and lung cancers (Sun *et al*, 2016; Zhu *et al*, 2017). Furthermore, ATG7 levels may also be of prognostic value in breast cancers patients (Desai *et al*, 2013). In individuals where inherited and somatic *ATG7* variants are discovered, preclinical mouse studies suggest that the genetic state of well‐characterised oncogenes and tumour suppressors should also be investigated. This may facilitate a deeper understanding of the role of ATG7 in these cancers—whether they underpin tumour growth or play a supportive role in the later stages of disease.

### Infection

Both autophagy and LC3‐associated phagocytosis are involved in the innate immune response that protects cells from invading pathogens (Levine *et al*, 2011; Heckmann *et al*, 2017). Although pathogens have evolved to modulate autophagic activity, even using it to enhance their pathogenesis, evidence in adult mouse models demonstrates that loss of *Atg7* increases infection susceptibility (Karsli‐Uzunbas *et al*, 2014). However, the role of ATG7 in human models of infection remains context‐specific. ATG7 restricts *mycobacterium tuberculosis* (Singh *et al*, 2006; Liu *et al*, 2020) and human papillomavirus infection (Griffin *et al*, 2013) and limits Chikungunya virus pathogenesis (Joubert *et al*, 2012). ATG7‐dependent autophagy is also stimulated upon infection with Influenza A, leading to endogenous presentation of epitope on MHC class II molecules (Deng *et al*, 2021). ATG7 also limits poliovirus infection; A defect in stimulus‐induced autophagy was observed in primary fibroblasts taken from a patient with poliomyelitis after polio infection, with exome sequencing identifying a heterozygous p.A388T (NP_006386.1) *ATG7* variant (Brinck Andersen *et al*, 2020). On the other hand, HIV‐1 hijacks autophagy to increase viral yield, before HIV protein Nef acts as an anti‐autophagic factor to prevent HIV degradation (Kyei *et al*, 2009). It has also been shown that autophagy selectively degrades Tat to restrict HIV‐1 infection (Sagnier *et al*, 2015). This autophagic dichotomy is observed in hepatitis C virus (HCV) infection, too. ATG7 inhibition suppresses HCV replication (Sir *et al*, 2008), but ATG7 activity enhances the innate immune response in HCV‐infected hepatocytes (Shrivastava *et al*, 2011). Consistent with a role in regulating inflammatory responses, inflammasome activity is enhanced in *Atg7* KO mouse infected with *Pseduomonas aeruginosa*, impairing pathogen clearance, thus implicating ATG7 in sepsis pathogenesis (Pu *et al*, 2017).

## Therapeutic approaches

Autophagy‐modulating therapeutics are of broad interest and have been studied in a number of settings (Fig 3). Evidence suggesting that dysfunctional autophagy contributes to neurodegenerative disorders has led to clinical studies assessing whether autophagy‐inducing compounds can improve neurological function and/or delay disease progression. In contrast, essential autophagic activity in cancer cells is predicted to maintain nutrient supply and prevent oxidative stress, thus contributing to treatment resistance. Hence, inhibition of autophagy may improve cancer treatment efficacy. The ability to modulate ATG7 activity directly using drugs may therefore have widespread implications. In cases where inherited ATG7 deficiency underlies disease, alternative approaches may be more beneficial.

### Treating ATG7 deficiency in neurological disorders

It is not unreasonable to anticipate that the number of patients identified harbouring biallelic, pathogenic *ATG7* variants will increase, and be associated with an ever‐widening spectrum of clinical presentations. The prevalent neurological phenotypes observed in the patients identified to date suggest that restoring autophagic function in nervous system would provide the optimal therapeutic approach. This is supported by analogous models of autophagy in Atg5‐deficient mice wherein neural expression of *Atg5* rescues perinatal lethality and extends life up to 8 months (Yoshii *et al*, 2016). Such an approach in human patients, for example using adeno‐associated viral gene therapy, has been widely investigated, but limitations must be overcome before this is a viable option (Wang *et al*, 2019a). This approach has, however, yielded success for the treatment of spinal muscular atrophy, a progressive motor neuron disease (Mendell *et al*, 2017). Bypassing the requirement of ATG7 for LC3 lipidation offers an alternative strategy. Infection with vaccinia, the live virus used in the smallpox vaccine, induces LC3 lipidation independently of ATG7 and ATG5 (Moloughney *et al*, 2011). Identifying the combination of viral and cellular factors driving non‐canonical LC3 lipidation under these circumstances could lead to the development of a viable therapeutic. In *Drosophila melanogaster*, Uba1 functions in an Atg7/Atg3‐independent autophagy pathway that is dependent on Atg8, but this has not been described in mammals (Chang *et al*, 2013). A different strategy would require a deeper understanding of the molecular events that lead to pathology. Studies using mice have demonstrated that liver injury in *Atg7*‐null mice can be remedied by *p62/SQSTM1*, *NFE2L2*, *Yap* or *p53* deletion, but the intracellular consequences of endogenous human *ATG7* inactivation in patient neural cells remain to be investigated (Komatsu *et al*, 2007a; Inami *et al*, 2011; Lee *et al*, 2018b; Yang *et al*, 2020). It would also be interesting to uncover whether ATG7‐independent degradation pathways may compensate for dysfunction of classical degradative autophagy. Of note, the RAB9‐dependent autophagy pathway, termed “alternative autophagy”, does not require ATG7 or LC3 lipidation, yet further work is required to understand both the molecular signatures and physiological importance of this pathway, although progress is being made (Nishida *et al*, 2009; Shimizu, 2018; Yamaguchi *et al*, 2020).

Preclinical mouse studies have suggested that enhancing ATG7 activity could help treat neurodegeneration (Donde *et al*, 2020). In human neurodegenerative conditions where defective autophagy is implicated, clinical trials have largely focussed on the use of compounds that activate autophagy. Some of these enhance autophagy via mTORC1 inactivation, including rapamycin (Mandrioli *et al*, 2018), idalopirdine (Wilkinson *et al*, 2014; Matsunaga *et al*, 2019) and SB‐742457 (Maher‐Edwards *et al*, 2010), whereas others (e.g. lithium (Sacca *et al*, 2015)) deliver remedy through TORC1‐independent mechanisms. Novel therapeutics that are able to enhance the selective delivery of cytoplasmic constituents (including mitochondria and mutant Huntingtin protein) to the autophagosome have also been reported (Li *et al*, 2019; Takahashi *et al*, 2019). It is unclear whether such approaches could meet the clinical demands of ATG7‐deficient patients.

### Targeting ATG7 to enhance cancer treatment

Although aberrant ATG7 activity is not known to commonly underpin human cancers, it has been theorised that disrupting autophagy can improve the potency of anti‐cancer therapeutics. A number of clinical trials have used blocker of autophagy hydroxychloroquine in combination with classical cancer therapies, yet results have been mixed (Mulcahy Levy & Thorburn, 2020). Combining autophagy inhibition with proteasomal inhibition has provided promise, with this approach leading to prostate cancer cell death (Zhu *et al*, 2010). Attenuating ATG7 function to sensitise tumour cells to cancer treatments has also been investigated in preclinical models with some success. ATG7 inactivation enhances the effectiveness of anti‐cancer therapeutics in lung and breast cancer cell treatment (Han *et al*, 2011; Desai *et al*, 2013; Yue *et al*, 2013). Moreover, endogenous noncoding RNA molecules miR‐17 and miR‐137 diminish *ATG7* expression, sensitising several cancer cell lines to chemotherapeutics or low ionising radiation (Comincini *et al*, 2013; Zeng *et al*, 2015). Modulating the *FoxO1/ATG7* axis may also provide a therapeutic opportunity. *FoxO1* encodes a tumour suppressor gene that drives cell death in human colon tumours via autophagy (Zhao *et al*, 2010). In bladder cancer, tumorigenic growth is also attenuated by FoxO1, yet this appears to be stimulated by ATG7 inhibition (Zhu *et al*, 2017). Indeed, the effectiveness of targeting ATG7 activity may be dependent on the genetic status of tumour suppressors and oncogenes. For example, whether autophagy promotes or reduces pancreatic tumour growth in mice appears to be dependent on p53 status (Rosenfeldt *et al*, 2013).

It is known that a number of cancer cell lines are highly autophagy‐dependent, and although they are generally resistant to loss of autophagic function (e.g. through ATG7 deletion), there is evidence that populations of cells are able to adapt to autophagy inhibition by upregulating different cellular pathways including NRF2 signalling (Towers *et al*, 2019). Interestingly, loss of LC3‐driven mitophagy via *ATG7* deletion upregulates formation of mitochondrial‐derived vesicles, 70–150 nm structures that laterally bud from mitochondria encapsulating selective cargo that can deliver material to the endolysosomal system for degradation, or to peroxisomes (Sugiura *et al*, 2014; Towers *et al*, 2021). These results offer druggable targets under circumstances where autophagy‐dependent cancers resist autophagy inhibition that attempts to enhance anti‐cancer treatments. They may also offer insight into how patients with inherited *ATG7* variants are able to survive into adult life. Overall, these findings reinforce the complexity underpinning human cancers and support the delivery of context‐specific therapeutic approaches, whilst the development of high specificity pharmacological inhibitors is also encouraging. This was demonstrated recently whereby Vps34 inhibition improved immunotherapy outcomes in preclinical models (Noman *et al*, 2020).

## Concluding remarks

It has now been demonstrated that inherited ATG7 deficiency causes congenital human disease hallmarked by neurodevelopmental deficits (Collier *et al*, 2021). Remarkably, humans can survive with mild–moderate neurological impairments despite undetectable levels of ATG7 protein. Future investigations will hopefully reveal which cellular pathways compensate for the absence of classical degradative autophagy, contributing to the survival of these patients. Recent advances suggest that mitochondrial‐derived vesicles and upregulated NRF2 signalling may be two mechanisms by which autophagy‐deficient cells are able to survive (Towers *et al*, 2019, 2021). Moreover, FIP200 clustering facilitates the bypass of LC3 lipidation in autophagy and promotes selectivity (Ohnstad *et al*, 2020).

Although the link between ATG7 and complex human disorders remains mostly elusive, the increasing associations between altered autophagy and complex human disorders suggest that directed modulation of ATG7 could provide a promising therapeutic opportunity. Studies in mice suggest that the most appropriate therapeutic approach when inherited ATG7 dysfunction underpins pathology may involve targeted neural restoration of ATG7 expression or alleviating downstream molecular consequences that may drive disease manifestation. In contrast, evidence suggests that disrupting autophagy may improve anti‐cancer therapeutics. Currently, inhibiting autophagy largely involves targeting the endolysosomal system leading to a broad impact on cellular homeostasis. Hence, there is a crucial need to develop selective autophagy inhibitors. Given that ATG7 has enzymatic activity, developing drugs that directly target ATG7 represents an attractive therapeutic strategy and may lead to more specific outcomes. For such approaches to be clinically feasible, it will be important to understand how these treatments affect the various biological pathways influenced by ATG7, and more broadly, how these pathways interact across cellular space and time.

Importantly, further analysis of ATG7‐independent degradation mechanisms will be key to understanding intracellular turnover in humans and whether these pathways can compensate under circumstances of autophagy dysfunction. The continued development of disease models and sensitive tools to monitor ATG7 activity *in vivo* will surely drive further progress in this exciting field.

## Conflict of interest

The authors declare that they have no conflict of interest.

### For more information

- OMIM: https://omim.org

**Pending issues**

- Delineating how the autophagy‐related and autophagy‐independent activities of ATG7 are regulated.
- Investigating how defective autophagy impairs neural integrity.
- Examination of how cells compensate for loss of classical degradative autophagy.
- Clarifying the role of ATG7 in complex disorders.
- Development of therapeutics that specifically target ATG7.
